# Sensing with Polarized LIDAR in Degraded Visibility Conditions Due to Fog and Low Clouds

**DOI:** 10.3390/s21072510

**Published:** 2021-04-03

**Authors:** Ayala Ronen, Eyal Agassi, Ofer Yaron

**Affiliations:** 1 Environmental Physics Department, Israel Institute for Biological Research (IIBR), Ness Ziona 7410001, Israel; eyala@iibr.gov.il; 2 Rafael Advanced Defense Systems, Haifa 3102102, Israel; ofery@rafael.co.il

**Keywords:** environment perception, computer vision, sensors, driver assistance, automatic control, optimization, robotics

## Abstract

LIDAR (Light Detection and Ranging) sensors are one of the leading technologies that are widely considered for autonomous navigation. However, foggy and cloudy conditions might pose a serious problem for a wide adoption of their use. Polarization is a well-known mechanism often applied to improve sensors’ performance in a dense atmosphere, but is still not commonly applied, to the best of our knowledge, in self-navigated devices. This article explores this issue, both theoretically and experimentally, and focuses on the dependence of the expected performance on the atmospheric interference type. We introduce a model which combines the well-known LIDAR equation with Stocks vectors and the Mueller matrix formulations in order to assess the magnitudes of the true target signal loss as well as the excess signal that arises from the scattering medium radiance, by considering the polarization state of the E–M (Electro-Magnetic) waves. Our analysis shows that using the polarization state may recover some of the poor performance of such systems for autonomous platforms in low visibility conditions, but it depends on the atmospheric medium type. This conclusion is supported by measurements held inside an aerosol chamber within a well-controlled and monitored artificial degraded visibility atmospheric environment. The presented analysis tool can be used for the optimization of design and trade-off analysis of LIDAR systems, which allow us to achieve the best performance for self-navigation in all weather conditions.

## 1. Introduction

In recent years, we have witnessed a rapid increase in R&D efforts for an adoption of active imaging sensing techniques for autonomous locomotion, driving, surveillance, and defense applications. One of the fastest growing technologies employed in this field is the use of small LIDARs for 3D sensing—mainly as a leading solution for autonomous vehicles (and maybe for drones at the next phase) (see, for example, the comprehensive reviews available elsewhere [1,2]). An active sensing technique consists an illuminating apparatus (typically laser-based, but not in necessarily (https://leddartech.com, accessed on 1 March 2021) over a large field of view adjoined with a collective device integrated with a fast response detector. Sophisticated analysis methods of the received signal enable a 3D reconstruction of the scanned area. The obtained 3D map is in turn manipulated in order to enable the platform to make a fast decision how to react and provide safe navigation within an ever-changing environment.

This task is challenging enough even in ideal conditions when the propagation medium has no effect on the quality of the received signal needed for the situation awareness sensor. However, because degraded visibility conditions are common, a series of important questions arises, such as:

1. What is the influence of the degraded medium on the quality of the 3D reconstructed picture? Can it be quantified as a function of the visibility range and the properties of the interfering media?
2. Does the polarization state of the LIDAR matter? What is the dependence of polarization in the type of atmospheric disturbance?
3. What is the minimum visibility range that a LIDAR sensor can tolerate for effective and safe autonomous driving?

The necessity and the importance of dealing with these questions for vision-based safety-related sensors were extensively discussed recently by Kim and Sumi [3]. However, although their work aimed to explore the fog as the environmental condition for a robot, they did not use a self-navigation platform with active illumination, and their imitation of an actual fog with nozzles produced much larger water droplets than those that actually exist in a real fog. Other recently published works have dealt in the estimation of the performance of LIDARs and other ADAS (Advanced Driver-Assistance Systems) techniques in adverse atmospheric conditions such as fog, rain, and snow [4,5,6,7]. However, they usually present empirical results of commercially available LIDARs that do not allow a raw signal analysis, without considering a signal scattering model. Furthermore, in most of the tests held in fog chambers, the size distribution of the “fog” was more “cloud”-like. A very good analysis of LIDAR performance in degraded environmental conditions is presented in Rasshofer et al. [8] and involves a basic scattering model. All of these studies did not treat the polarization state of the signal. There was no comprehensive work that combined a detailed analytical model, controlled measurements in a fog chamber with several size distributions and analysis of the influence of the polarization state. In order to complete the treatment for active platforms, the main aim of our work is to explore, both theoretically and experimentally (in a well-controlled and monitored environment), the performance of autonomous LIDAR platforms in degraded atmospheric conditions of fog and low clouds and to assess whether the use of the polarization state of the LIDAR can reduce some of the atmospheric interferences.

**Figure 1:**
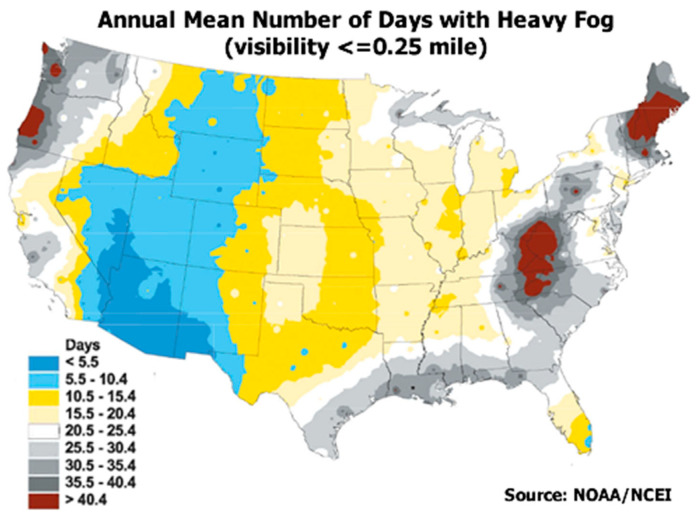
Annual mean number of days with heavy fog over the continental United States [10].

The most frequent degraded visibility weather conditions on the ground level are due to fog and low clouds. By definition, a fog reduces the visibility range down to less than 1 km [9]. The frequency of low visibility events has a complex dependence on the topographical properties of a specific site, synoptic conditions, time of day, the geographical region, and additional factors. Does the frequency of degraded visibility conditions justify the mitigation efforts? An observation of the frequency of fog events across the continental United States (Figure 1), shows clearly that this is indeed the case.

The reduction in LIDAR performance arises from interactions of the emitted and reflected electromagnetic radiation with the ambient aerosols, resulting in absorption and scattering processes that change the properties of an electro-magnetic wave, including its polarization state. The obtained signal in a LIDAR receiver (or at any typical imaging sensor in the same scenario) is a sum of two contributions: scattered light from the aerosol medium, which arrives from all directions and ranges, and the signal returned by the solid target at the end of the line of sight (LOS), attenuated by the atmosphere. An autonomous platform is expected to fail to operate either if the reduction in the signal-to-noise ratio will fall below a preset threshold, preventing it from the detection of a legitimate target, or by false interpretation of the scattered atmospheric signal as a true target.

Therefore, it is clear that fog and cloudy conditions, which are characterized by extremely low visibility ranges, should be considered a serious problem for the assimilation of the use of LIDAR systems into autonomous mobile platforms. Consequently, it requires an estimation of their performance limitations. It becomes critical to assess the influence of severely degraded atmospheric conditions on the performance of autonomous vehicles and to establish efficient methods to mitigate their effect.

The theoretical approach that is introduced in the second section of this article is based on the formulation of the returned LIDAR signal by the well-known “LIDAR equation” [11], expanded to consider the polarization states of the E–M waves based on the Mie scattering model [12].

The issue of polarization is important in this context for two reasons. Firstly, from geometrical considerations: in the case of common real scenarios, the LIDAR source and the receiver are not always coinciding. Those non-coaxial transfers lead to depolarization that should be treated carefully and properly. Secondly, an important property of Mie scattering is that the depolarization is zero only in the unique angles of 0° and 180°, so it is relevant only for perfect backscattering scenarios. This polarization effect is a well-known phenomenon in clouds [13,14], and the purpose of the present study is to investigate its influence on LIDAR performance in degraded visibilities. Special care was given to testing our theoretical estimates in a well-controlled and monitored environment that imitates as best as possible true atmospheric foggy conditions.

It is worth noting recent work [15] which explored the polarimetric LIDAR backscattering contrast of linearly and circularly polarized pulses for ideal depolarizing targets in generic water fogs. However, ideal depolarizing targets cannot be guaranteed in self-navigation scenarios, especially in an urban environment, and their experimental work was based on imitating an actual fog with nozzles.

Our model involves the geometrical considerations needed to calculate the scattering processes for light scattering which depends on the geometry and is not a simple back scattering scenario (which was the only scenario that has been widely considered so far). We will show that for this case, the use of a simple single scattering method fails to predict the interplay between the returned and atmospheric scattered contributions to the total signal at the receiver, which might occur in common real scenarios.

The third section will describe our experimental setup. It was not feasible to conduct well-controlled tests in an open atmosphere, and because experimental work requires a stable and homogenous environment, we therefore measured and characterized LIDAR performances inside an aerosol chamber. Such facilities provide well-defined degraded atmospheric environments, with a wide range of droplet size distributions and visibility ranges [6,16,17]; for example, a previous study has successfully been conducted in our fog chamber [18]. This section also presents the experimental results adjoined with a comparison to the expected radiative transfer calculations results according to our proposed model. The results show good agreement between the two, and clearly support our hypothesis that using the correct configuration of the polarization state can significantly reduce the atmospheric degradation effect on the performance of a LIDAR system. Therefore, this technique might recover some of the performance loss of such systems for autonomous platforms in low visibility conditions. We will conclude, discuss, and summarize the work in the last section.

## 2. A Single Scattering Angle and Polarization Dependent Model for LIDARs

**Figure 2:**
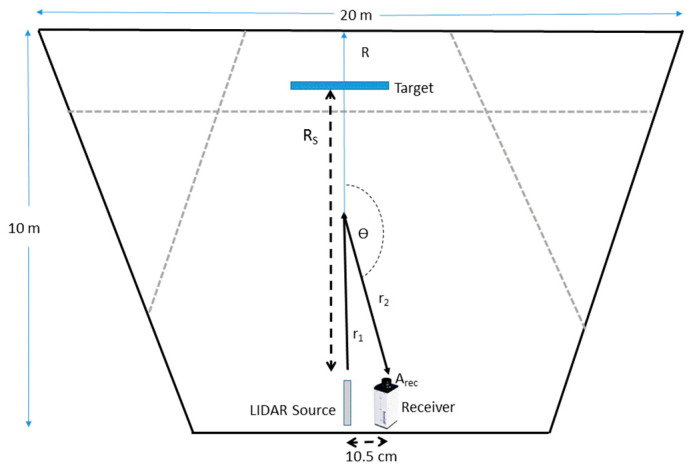
Top view of the model and corresponding experimental setup. Target location is seen along with single scattering trajectory where the receiver location is slightly shifted relative to the LIDAR source, and a scattering angle of θ ≠ 180° is obtained. The dashed grey lines represent the path of the 3 transmission measurements inside the aerosol chamber (see technical details in Section 3).

In this section, we will introduce an analytical model for calculating the detected single scattering signal for a LIDAR-like system operating in a dense scattering medium, including the polarization components. The predicted results will serve us as a basis for a comparison with actual measurements which will be presented in the next section. We will start our treatment by following the work of Kavaya et al. [19], who performed a detailed analysis of the returned signal from a solid target located inside the LIDAR’s FOV (field of view), relative to the atmospheric single-scattered signal. Consider a LIDAR beam which is pointed on a target located at a range of R_S_, as sketched in Figure 2.

A collinear transmitter–receiver geometry is assumed; thus, both the transmitting and receiving telescopes subtend small, but similar, solid angles. If the transmitted pulse starts at time *t* = 0 and ends at *t = τ*, and if we assume that the target subtends a larger FOV than the transmitted illumination, then the received signal power (*P*), range (*R*) dependency, is given by:

(1) $$P_{T}\left(R\right)=P_{t}\left[\frac{2}{c}\left(R-R_{S}\right)\right]\rho^{*}\frac{A_{rec}}{R_{S}^{2}}\eta_{sys}O\left(R_{S}\right)\exp\left(-2\alpha_{ext}CR_{S}\right)$$

where *c* is the speed of light, and *2R_S_/c* is the round-trip transit time of light. The term $P_{t}\left[\frac{2}{c}\left(R-R_{S}\right)\right]$ refers to the fact that a reflection from a solid target at the end of the *LOS* keeps the emitted pulse width and it corresponds to its distance from the source. $\rho^{*}$[sr−1] denotes the target reflectance, defined as the reflected power per steradian towards the receiver, divided by the incident power. It is common to consider $\rho^{*}$ for normal incidence as a Lambertian target, and therefore ρ*=ρrefπ, where $\rho_{ref}$ is the target reflectivity, determined by the material type, color and surface roughness. $A_{rec}$ is the effective receiver area, and $\frac{A_{rec}}{R_{S}^{2}}$ is the solid angle (sr) at the target subtended by the receiver. $\eta_{sys}$ is the system’s optical efficiency and *O(R_S_)* is the range-dependent overlap function, which approaches unity after a relatively short distance. If the target area (*A_Tar_*) is smaller than the illuminated area (*A_il_*), the factor (*A_Tar_/A_il_*) is added to the target signal [20]. $\alpha_{ext}$ is the atmospheric total mass extinction coefficient (m^2^·g^−1^), including scattering and absorption contributions, assumed to be constant along the trajectory, and *C* is the atmosphere concentration (g·m^−3^).

The last expression in Equation (1) is the integrated two-way attenuation by the atmospheric extinction as the emitted signal propagates from the source to the target and back. For poly-dispersed spherical particles with radius *r*, size distribution function *n(r)*, and density ρ, the mass extinction coefficient is:

(2) $$\;\alpha_{ext}=\frac{\int^{}\pi r^{2}Q_{ext}n\left(r\right)dr}{\int^{}\frac{4}{3}\pi r^{3}\rho n\left(r\right)dr}$$

where the extinction efficiency *Q_ext_* is the solution of the Mie model [12] and depends on the refractive index and on the sensor wavelength λ.

It is assumed that the receiver optics is small enough, so the forward scattered light collected by its optics is relatively small.

Due to the atmospheric interference, the overall power obtained at the receiver is the sum of the attenuated returned target signal, as defined in Equation (1), and the power that arrives as a result of the scattering processes in the atmospheric medium. In order to avoid adding unnecessary complexity, we will refer to the first order of scattering processes only, although the general approach hereafter can be readily extended to higher orders of scattering. The single scattering expression is obtained in a similar manner to the target signal, by considering the same LIDAR system characterized in Equation (1) directed into the atmosphere, without a target in its line of sight. For this scenario, the returned signal at time *t* is due to the total contributions of slabs of the atmosphere, each one of them at a thickness *cτ/2*, where *τ* is the pulse duration, centered at *R = c(t/2 − τ/4).* Note that unlike Equation (1), the returned light from the atmosphere is not limited to the time frame of $\frac{2}{c}\left(R-R_{S}\right)$, but is now continuous. The receiver dimensions are typically small compared to the range *R*; therefore, mainly scattered radiation at directions close to 180° will strike upon it and will significantly contribute to the detected signal. For a transmitted rectangular monostatic single-wavelength pulsed LIDAR with instantaneous power *P*_0_, the received power due to aerosol backscatter is given by the common form of the single scattering LIDAR equation:

(3) $$P_{S}\left(R\right)=\frac{c\tau }{2}\beta \left(R\right)C\frac{A_{rec}}{R^{2}}\eta_{sys}O\left(R\right)\exp\left(-2\alpha_{ext}CR\right)P_{0}$$

where *β* is the aerosol backscattering coefficient (m^2^ ·g^−1^ ·sr^−1^), defined as the fraction of incident energy scattered in the backward direction per unit solid angle per unit atmospheric range and concentration. Let us assume that *β* is constant, which is reasonable assumption for ground level sensing at short ranges.

In several applications, the source and receiver spots do not always coincide, and therefore the scattering angle differs from 180°, and the light trajectory is not a round trip. In this case, the single scattering expression, Equation (3), has to be expanded by considering the dependence on the scattering angle, ranges, and polarization states. For this geometry, we will follow the theoretical model presented in Kleiman and Shiloah [21] that was developed for the case of multiple scattering by particles of a laser beam that propagates through the atmosphere. The model is based on the well-described Mie E–M wave scattering model. According to this approach, the transformations of the light irradiance are represented by operation of the Mueller scattering matrix on the “Modified Stokes vector” [22] of the incident light. The Modified Stokes vector is a version of the Stokes vector, suitable to polarization processes and is defined as:

(4) $$I=\left[I_{II},I_{t},U,V\right]$$

where *I_II_* and *I_t_* are the parallel and perpendicular polarization components of the electromagnetic vector of the incident light, respectively, and *U* and *V* describe the circular polarization. The Mueller scattering matrix, which is also known as the phase-function of a spherically symmetrical particle, for the Modified Stokes vector, is defined with the intensity functions as follows:

(5) $$M_{Mie}=\begin{array}{cccc} i_{2} & 0 & 0 & 0\\ 0 & i_{1} & 0 & 0\\ 0 & 0 & i_{3} & -i_{4}\\ 0 & 0 & i_{4} & i_{3} \end{array}$$

In a similar manner to Equation (2), the scattering matrix becomes:

(6) $$M\left(\theta \right)=\frac{\int^{}\pi r^{2}M_{Mie}\left(\theta ,\lambda \right)n\left(r\right)dr}{\int^{}\frac{4}{3}\rho \pi r^{3}n\left(r\right)dr}$$

The refractive index of water [23] was used to calculate the Mie coefficients [24]. In *λ* = 632 nm, the refractive index is 1.3317 + 1.46 × 10^−8^ × *i*. The imaginary part is relatively small compared to the real one; therefore, the extinction of a travelling light through the foggy or cloudy atmosphere is mainly due to the scattering processes and the absorption contribution may be neglected.

The size distribution of droplets in clouds and fog has been extensively studied for several decades. Deirmenjian [25] proposed commonly accepted typical density size distributions of cloud droplets as a function of their radii. Additional size distributions based on other works can be found, for example, in Arnott et al. and Liu et al. [26,27]. Local fog measurements have been reported in the literature. Measurements of droplet sizes of a fog were presented in the work of Price [28]. A classification of 160 fog samples droplet size distributions was presented in Podzimek [29], showing a wide range of typical droplets sizes. Ground level fog events were described in detail in the work of Thies et al. [30], which surveyed measurements of droplet size distributions of radiation fog in Germany.

**Figure 3:**
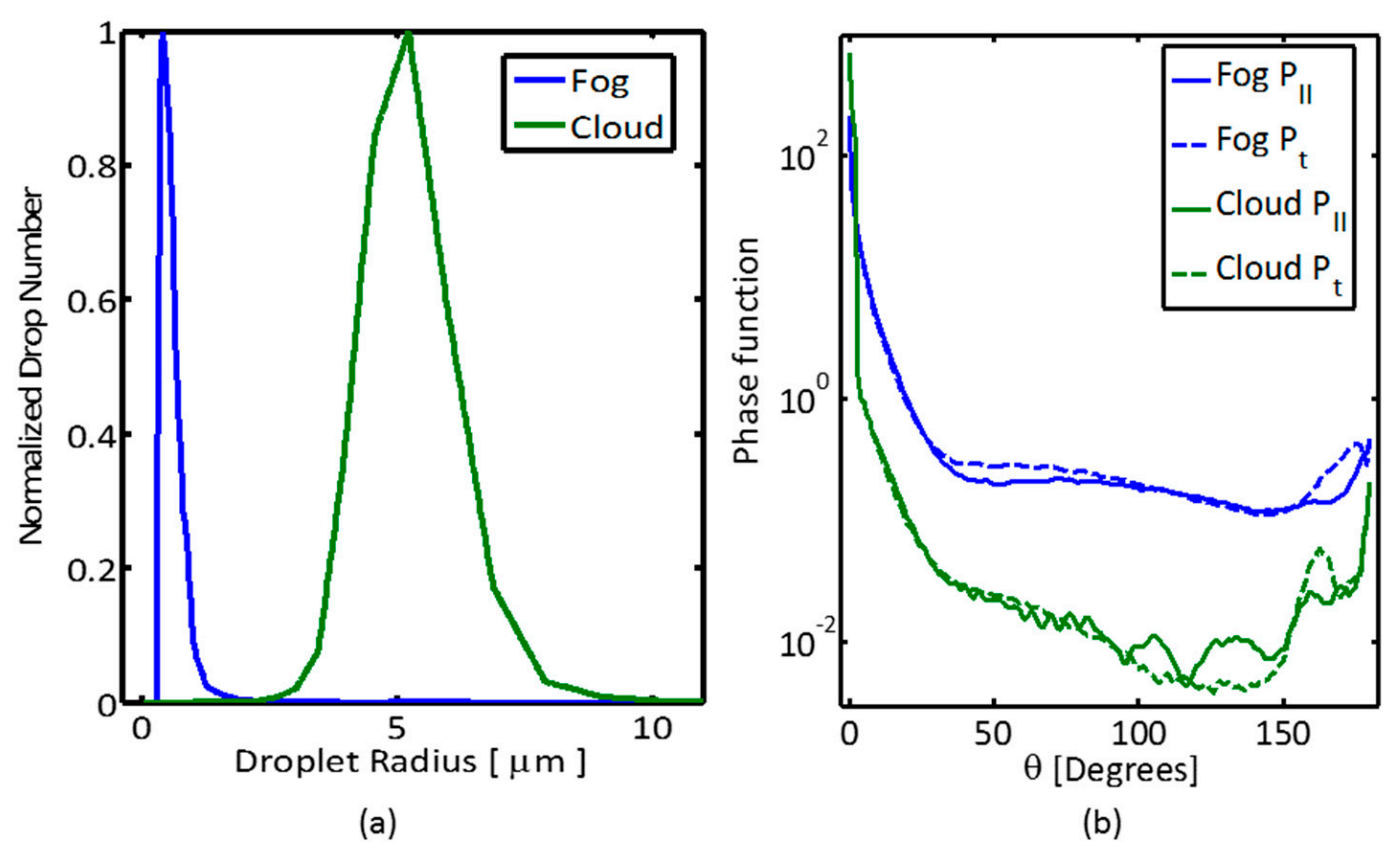
(**a**) Droplet size distributions of artificial cloud and fog hydrosols dispersed during the aerosol chamber experiments. (**b**) The phase function component angle dependences at λ = 632 nm that correspond to the distributions on the left-hand side (log-scale). The two polarization components, *P_II_*—parallel and *P_t_*—the orthogonal, are represented by full and dotted lines, respectively.

The data that have been described in these publications served as a guide for the aerosol generators used in our experiments. Figure 3 shows the measured droplet size distributions and the corresponding calculated parameters of the phase function of our artificial “fog-like” and “cloud-like” degraded visibility atmospheres that were dispersed in the chamber tests, and their results are presented in the next section.

It can be seen from Figure 3 that the two different aerosol media have distinctive droplet size distributions. The “fog-like” scattering intensity is larger than that of the “cloud-like” one, because fog droplet sizes are closer in scale to the light wavelength, and hence the radiation transfer is within the size parameter values of the “Mie regime”. We can discern that scattering angles of 0° and 180° are unique points where both polarization components are equal, with a depolarization of zero.

These polarization effects may be useful to reduce the negative atmospheric effect and can be used to block the unwanted backscattered radiation component. If a LIDAR emits linearly polarized light, then in an ideal case there should not be any returned signal in the perpendicular polarization direction [13], and the system could be durable against false returns. For scattering angles different from 180° (as we will show in the third section describing the experimental setup), some scattered light will contribute to the returned signal in the perpendicular direction, and therefore, the effect is somewhat limited, and its strength depends on geometry and visibility range.

In order to explore the polarization effect, a detailed radiative transfer solution is needed to track the change in polarization components. As mentioned before, the proper tool needed to accomplish this task is an expression of the E–M field in a Modified Stokes vector form, followed by an operation of the proper Mueller matrices.

Rotating the Stokes vectors relative to the scattering plane orientation is carried out by multiplying the rotational matrix *L*, which is the Mueller matrix for rotation of the polarization plane at an angle of *φ*, relative to the initial polarization plane:

(7) $$L\left(\varphi \right)=\begin{array}{cccc} cos^{2}\left(\varphi \right) & sin^{2}\left(\varphi \right) & sin^{2}\left(\varphi \right)/2 & 0\\ sin^{2}\left(\varphi \right) & cos^{2}\left(\varphi \right) & -sin^{2}\left(\varphi \right)/2 & 0\\ -sin\left(2\varphi \right) & sin\left(2\varphi \right) & cos\left(2\varphi \right) & 0\\ 0 & 0 & 0 & 1 \end{array}$$

The first order scattering signal at a scattering angle θ is obtained by applying the matrix L(−ϕ)·M(θ)·L(ϕ) on the initial Stokes vector, as shown in Equation (8):

(8) $${\bar{I}}_{S}\left(R,\phi \right)=\frac{c\tau }{2}\frac{A_{rec}}{r_{2}^{2}}C\eta_{sys}O\left(r_{2}\right)\exp\left(-\alpha_{ext}C\left(r_{1}+r_{2}\right)\right)\left[L\left(-\phi \right)\cdot M\left(\theta \right)\cdot L\left(\phi \right)\right]{\bar{I}}_{0},$$

where *r*_1_ and *r*_2_ are the lengths of light trajectories in the case of a scattering angle different from 180° (see Figure 2).

The elements of the Stokes vector on two sides of the equation can be transformed into units of power. As mentioned above, the first two elements of the Stokes vector are the parallel and perpendicular linear polarization irradiance components. In cases where the receiver is a camera, as it was in our experiment, no gating or range-dependence of the signal is essential, and the total power can be degenerated to the sum of all range-dependent signals:

(9) $$I_{S}\left(\phi \right)=\int^{}I_{S}\left(R\right)dR$$

Although the formulation which was introduced in Equations (7)–(9) is general, it should be evaluated and used with respect to a specific geometrical scenario. Therefore, let us demonstrate its practical use on the same geometry as was in our aerosol chamber measurements. Consider an experimental setup shown in Figure 2, where a laser source and a camera that serves as a sensor, do not coincide in their locations. The source is considered to be fully polarized, characterized by a Modified Stokes vector of [1; 0; 0; 0] (we assume a negligible coherent length).

For practical reasons, it is easier to describe atmospheric attenuation in terms of visibility range. The transmittance itself can be measured easily using a transmissometer. The visibility range is defined as the range at which the atmospheric transmittance falls to 2% (Koschmieder’s equation [31]):

(10) $$VisibilityRange=\frac{\ln\left(50\right)}{\alpha_{ext}C}$$

We have concluded the parametrization of Equations (7)–(9): sensor dependent terms (*P_0_*, *A_rec_*, etc.); geometrical setting (*r*_1_, *r*_2_); characterization of atmospheric medium through Equation (10), which in turn depends on the density and size distribution of the hydrosol cloud; and an E–M power manipulation by rotation of the Mueller matrix.

**Figure 4:**
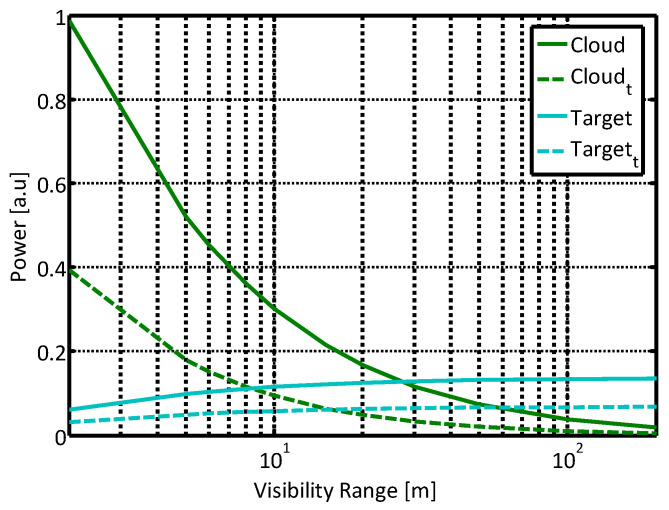
Calculated path integrated single scattering and target reflected power as a function of visibility, inside a “cloud-like” atmosphere, for un-polarized and polarized (marked by *t*) signals. Target location is 3 m from the sensor.

Figure 4 shows the calculated power obtained of the un-polarized and orthogonal polarization states inside a “quasi-cloud” environment, and the corresponding attenuated target signal. All signals were normalized, to eliminate the dependence in geometrical aspects of both sensor and set-up parameters such as the laser pulse duration, the receiver optical efficiency, and target characteristics, including reflectance and size.

As mentioned above, the signal obtained in the presence of an object in the sensor FOV is the sum of the target signal and the atmospheric scattering signal contribution, both shown in Figure 4. We can observe that using a polarized scattered signal, orthogonal to the initial polarization direction, reduces the obtained scattered signal and may decrease the atmospheric perturbation added to the target signal. For a perfect backscatter phenomenon, this orthogonal direction should be zero; for example, in configurations of 3D LIDARS used in autonomous driving applications.

Note that contrary to the atmospheric induced signals, the ratio between contributions of the unpolarized and polarized signals by the target return does not depend on visibility conditions, because the target is assumed to be Lambertian with no degree of polarization. Thus, employing the polarization effect by blocking the parallel component of the signal will not be followed by a complete elimination of the desired target signal. It should be emphasized that our approach is general, and other target reflectance properties can readily be incorporated in our model.

**Figure 5:**
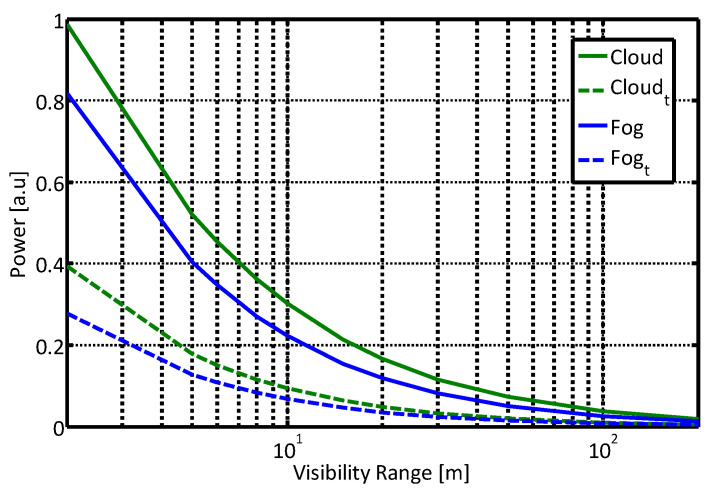
The calculated path-integrated single scattering power as function of visibility inside cloud-like and fog-like hydrosol clouds, for un-polarized and polarized (marked by *t*) signals.

Different atmospheric aerosols change the respective magnitude of the received signals, as can be seen in Figure 5. The plot shows the calculated power of unpolarized and orthogonal components of the scattered light for different hydrosol media, the “cloud-like” and the “fog-like” conditions. As can be expected, the larger “cloud-like” droplets returned much larger power than the smaller ones of “fog-like” droplets. We will experimentally support this theoretical conclusion in the next section.

## 3. Experiment and Results

Conducting well-controlled validation tests for the proposed approach is a challenging task because of several reasons. This is due to the complexity of the problem and the dependence of the received signal on many input parameters of the sensor (which itself is not a simple one), of the environment, and the fact that the figure of merit of the whole system is somewhat elusive and subject to many trade-offs. For example, should we consider scarifying some of the good weather performances for a better one in degraded visibility conditions? For this reason, we have decided to focus on keeping a well-defined and monitored hydrosolic atmospheric environment in our first experiments. We expected that this path would enable us to explore the measurement results with respect to the different hydrosol environments, in which the polarization state serves as a well-defined validation test parameter. Furthermore, we estimated that the ability to create a reliable and monitored hydrosolic environment is an essential key factor for any future extended tests.

**Figure 6:**
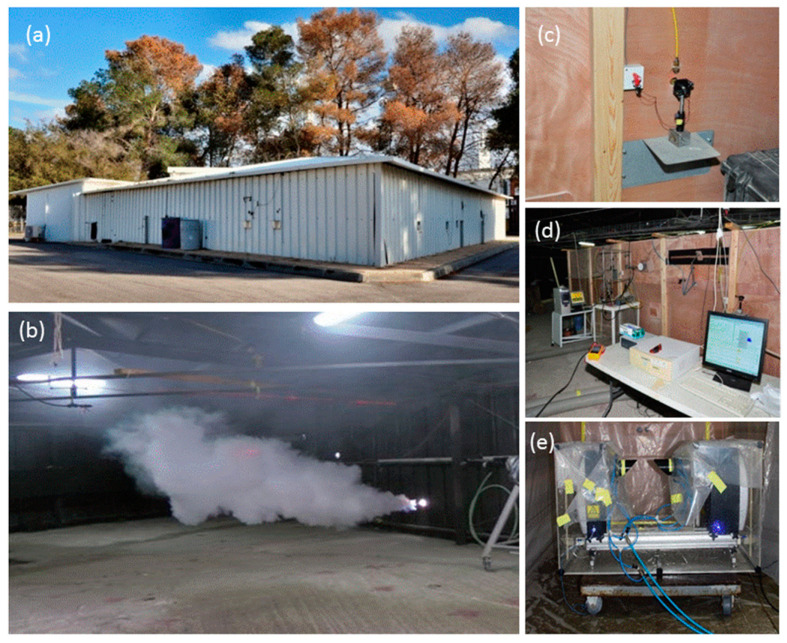
(**a**) Israel Institute for Biological Research (IIBR) aerosol chamber. (**b**) Initial filling of an artificial cloud into the chamber. (**c**) External view of one of the diodes that served for transmission measurements. (**d**) External chamber wall, showing remote control instrumentation. (**e**) Spraytec instrument for measuring the droplet size distribution in real-time.

Our experiment was held inside aerosol chamber at the Israel Institute for Biological Research (IIBR), a facility which is used for the evaluation of Electro-Optic sensors performance in challenging atmospheric conditions. It is a 450 m^3^ triangle hangar with sides of about 10–20 m and 2.5 m in height (see Figure 2). It allows the formation of artificial atmospheric conditions, including hydrosols (cloud and fog), smoke, and dust and oil droplets, in which all characterizing parameters of the indoor environment are carefully maintained by remote control. The internal atmosphere is homogenized using a series of powerful fans. The concentration and visibility uniformity of the aerosol environment inside the chamber is monitored continuously by measuring the transmittance across the chamber at three different lines of sight by self-built optical transmissometers. Each one of these transmissometers consists of a laser diode (ThorLabs, Newton, NJ, USA) at a wavelength of 638.3 nm and two photo-detector channels for the transmitted signal and the clear reference. The instantaneous droplets size distribution inside the chamber is monitored by the laser diffraction system, “Spraytec” (Malvern, UK). Some figures that show the chamber are presented in Figure 6.

The chamber was filled with two types of artificial atmospheres, simulating cloud and fog. “Cloudlike” droplets were injected into the chamber with several ultrasonic aerosol generators. The “fog-like” environment was produced by fog machines. The experiment illumination source was a 13 W projector with an elliptical 26° × 8° beam. Two sensors measured the returned signals. The first sensor was a JAI camera with 25° field of view, and the second was a TE cooled digital EMCCD camera.

**Figure 7:**
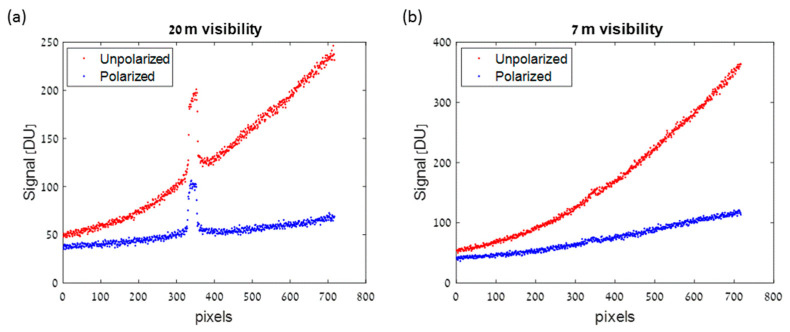
Received video line signal at polarized, (blue line) and unpolarized (red line) cameras. The video line contains a black background with a white object at the middle of the frame. The target width (about 8 cm) corresponds to 23 pixels. (**a**) At cloud visibility range of 20 m, the white object can be clearly seen. (**b**) Taken at a visibility range of 7 m, the object cannot be observed despite the very similar received cloud signal at the polarized camera.

The JAI camera was operated with and without a polarizer. For the unpolarized tests, a 1.5 ms integration time was set. For the polarized test, 7 ms was chosen, in order to compensate for the reduced illumination power and the receiver polarizer. The camera field of view as well as the projector illumination beam shape were chosen to match the physical size of the fog chamber so adjacent walls would not be illuminated. Figure 7 shows the analyzed return at a single detector line for two different visibility ranges. The setup included a narrow white board located 6.1 m from the sensor (see Figure 2). The return signal of the un-polarized test was multiplied by integration times ratio of the two tests. It can be seen that the backscatter signal of the polarized setup is significantly reduced. The larger backscatter on the right side of this cross-section resulted from the geometrical setup, in which the projector was located to the right of the sensor. The backscatter level at 7 m visibility was only slightly increased with respect to the 20 m visibility. However, the signal from the target at 7 m visibility was almost invisible. The forward and backward attenuation for a path length which is very close to visibility range reduced the received signal to below noise level. Figure 7 shows the analyzed return from the detector in two different visibility ranges; for a setup where a white target is located 6 m from the sensor, see Figure 2.

**Figure 8:**
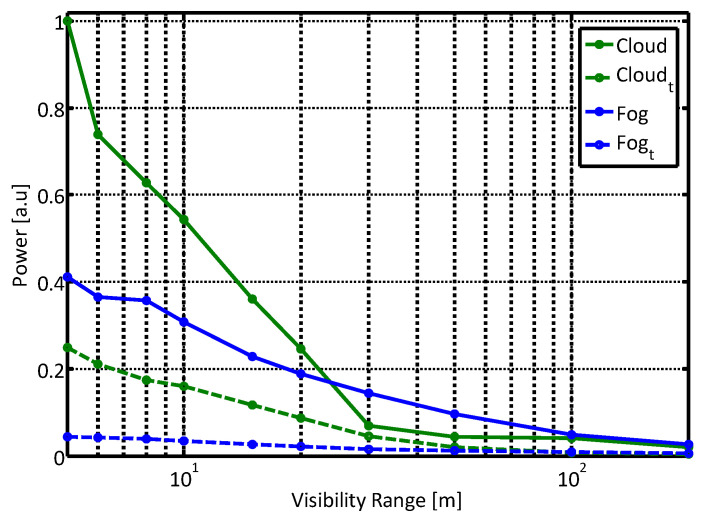
Normalized measured power, un-polarized and polarized (marked by the subscript *t*) signals. Aerosol chamber was filled with artificial fog and cloud.

The second sensor, a TE cooled digital EMCCD camera (“Sensicam” by pco., Kelheim Germany) served as a sensitive and high dynamic range sensor, for measurements of the scattered signals in a constant exposure time. The usage of a camera as a detector enabled simultaneous measurements at multiple angles with respect to the laser direction. A linear polarizer was set in front of the camera optics, in perpendicular direction to the polarized emitted intensity. It enabled us to compare measurements of the whole signal with measurements of the perpendicular return only. Figure 8 shows the total measured power obtained with the Sensicam sensor, at the unpolarized state and at the orthogonal polarization direction, in “cloud” and “fog” conditions. The signal was obtained by summing all the pixels in the camera with a uniform integration time (75 ms) at all scenarios. The constant dark background bias was subtracted from all the obtained data.

Comparing the measured data presented in Figure 8 with the model results presented in Figure 5 shows a good agreement. The first order polarization model describes the measured intensity in both parallel and perpendicular polarization directions very well. It strongly supports our hypothesis that it is possible to employ the polarization state in order to reduce the path scattered radiance in a degraded visibility environment. Furthermore, it enhanced our confidence that the proposed model is suitable for the more general scenario of gated sensing. We plan to explore this topic in our next experimental phase.

## 4. Summary and Conclusions

A model for the expected LIDAR performance degradation in low visibility atmosphere and its dependence in the atmospheric hydrosolic type was introduced. The use of such a model is essential to estimate the expected performance of LIDAR-based autonomous platforms. It also can be used as an efficient tool for trade-off system design and engineering analysis for all weather optimized operation. The model was derived from the classic LIDAR equation combined with Modified Stokes vectors and Mueller matrix formulation that extended the capability of the LIDAR equation to evaluate the LIDAR’s performance in severely degraded visibility environments. One of the benefits of its use is that it enabled assessment of the utility of the polarization state as a tool to mitigate some of the effects of the atmospheric scattered light on the LIDAR performance. The theoretical analysis showed that using orthogonal polarization directions (emitter and sensor) significantly reduces the intensity of the scattered light relative to a valid target return signal.

In order to validate our theoretical results, well-controlled and monitored tests were performed inside an aerosol chamber in an artificial environment of dense cloud and fog. The properties of these artificial cloudy environments were carefully adjusted as much as possible in order to imitate the true size distribution of natural cloud and fog types that are widely accepted. Our first measurements emphasized the establishment of a reliable and realistic artificial low visibility atmosphere. We believe that it is a key and essential factor which must be well-proven before any conduction of advanced tests in order to prevent false conclusions. Therefore, in our first measurements we focused on a simple experimental setup whose main aim was to support/disprove our polarization states analysis. The experimental results showed a good agreement with the quantified predictions of the theoretical model. These results clearly show that the use of a polarized source together with a cross-polarized receiver can improve the target-signal to atmospheric-signal ratio in a dense aerosol medium for a LIDAR system. Therefore, implementing polarimetric imaging techniques in LIDARs can enhance the performance of autonomous vehicles in poor visibility conditions. The importance of this tool has a much wider aspect than a mere single possible solution for the mitigation of low visibility atmospheric effects. It can be considered a general and rigorous tool for optimized design and trade-off analysis of self-navigated systems which allow us to achieve the best performance in all weather conditions. After proving that our setup can support the model validation, we intend to extend our tests in the future for more complex and relevant scenarios.

## References

[1] Royo S., Ballesta-Garcia M. (2019). An Overview of Lidar Imaging Systems for Autonomous Vehicles. Appl. Sci..

[2] Guan H., Li J., Cao S., Yu Y. (2016). Use of mobile LiDAR in road information inventory: A review. Int. J. Image Data Fusion.

[3] Kim B.K., Sumi Y. (2020). Vision-Based Safety-Related Sensors in Low Visibility by Fog. Sensors.

[4] Bijelic M., Gruber T., Ritter W. A benchmark for lidar sensors in fog: Is detection breaking down?. Proceedings of the 2018 IEEE Intelligent Vehicles Symposium (IV).

[5] Judd K.M., Thornton M.P., Richards A.A. Automotive sensing: Assessing the impact of fog on LWIR, MWIR, SWIR, visible, and lidar performance. Proceedings of the Infrared Technology and Applications XLV.

[6] Kutila M., Pyykönen P., Holzhüter H., Colomb M., Duthon P. Automotive LiDAR performance verification in fog and rain. Proceedings of the 2018 21st International Conference on Intelligent Transportation Systems (ITSC).

[7] Kutila M., Pyykönen P., Holzhüter H., Colomb M., Duthon P. LIBRE: The multiple 3D LiDAR dataset. Proceedings of the 2020 IEEE Intelligent Vehicles Symposium (IV).

[8] Rasshofer R.H., Spies M., Spies H. (2011). Influences of weather phenomena on automotive laser radar systems. Adv. Radio Sci..

[9] (2005). Federal Meteorological Handbook Number 1: Chapter 8—Present Weather (PDF).

[10] Midwestern Regional Climate Center. https://mrcc.illinois.edu/living_wx/fog/index.html.

[11] Hey J.D.V. (2015). Theory of Lidar. A Novel Lidar Ceilometer.

[12] Bohren F., Huffman D.R. (1998). Absorption and scattering by a sphere. Absorption and Scattering of Light by Small Particles.

[13] Sassen K., Liou K.N. (1979). Scattering of polarized laser light by water droplet, mixed-phase and ice crystal clouds. Part I: Angular scattering patterns. J. Atmos. Sci..

[14] Sassen K. (2006). Polarization. LIDAR.

[15] Tremblay G., Roy G. (2021). Polarimetric LiDAR backscattering contrast of linearly and circularly polarized pulses for ideal depolarizing targets in generic water fogs. Appl. Opt..

[16] Kim B.K., Sumi Y. Performance evaluation of safety sensors in the indoor fog chamber. Proceedings of the 2017 IEEE Underwater Technology (UT).

[17] Trickey E., Church P., Cao X. (2013). Characterization of the OPAL obscurant penetrating LiDAR in various degraded visual environments. Degraded Visual Environments: Enhanced, Synthetic, and External Vision Solutions.

[18] Golovachev Y., Etinger A., Pinhasi G.A., Pinhasi Y. (2019). Propagation properties of sub-millimeter waves in foggy conditions. J. Appl. Phys..

[19] Kavaya M.J., Menzies R.T., Haner D.A., Oppenheim U.P., Flamant P.H. (1983). Target reflectance measurements for calibration of lidar atmospheric backscatter data. Appl. Opt..

[20] McManamon P. (2012). Review of ladar: A historic, yet emerging, sensor technology with rich phenomenology. Opt. Eng..

[21] Kleiman M.M., Shiloah N. (1999). Effect of dense atmospheric environment on the performance of laser radar sensors used for collision avoidance. Laser Radar Technology and Applications IV.

[22] Chandrasekhar S. The Equation of Transfer. Radiative Transfer.

[23] D’Almeida G.A., Koepke P., Shettle E.P. (1991). A Global Aerosol Model. Atmospheric Aerosols: Global Climatology and Radiative Characteristics.

[24] Mätzler C. MATLAB Functions for Mie Scattering and Absorption. https://omlc.org/software/mie/maetzlermie/Maetzler2002.pdf.

[25] Deirmendjian D. (1969). Single Scattering on Many Particles. Electromagnetic Scattering on Spherical Polydispersions.

[26] Arnott W.P., Schmitt C., Liu Y., Hallett L. (1997). Droplet size spectra and water-vapor concentration of laboratory water clouds: Inversion of Fourier transform infrared (500–5000 cm^−1^) optical-depth measurement. Appl. Opt..

[27] Liu Y., Laiguang Y., Weinong Y., Feng L. (1995). On the size distribution of cloud droplets. Atmos. Res..

[28] Price J. (2011). Radiation fog. Part I: Observations of stability and drop size distributions. Bound. Layer Meteorol..

[29] Podzimek J. (1997). Droplet concentration and size distribution in haze and fog. Studia Geophys. Geod..

[30] Thies B., Egli S., Bendix J. (2017). The Influence of Drop Size Distributions on the Relationship between Liquid Water Content and Radar Reflectivity in Radiation Fogs. Atmosphere.

[31] Middleton W.E.K. (1952). The visual range of objects in natural light. Vision through the Atmosphere.

